# Natural Phytochemicals as Novel Therapeutic Strategies to Prevent and Treat Parkinson's Disease: Current Knowledge and Future Perspectives

**DOI:** 10.1155/2021/6680935

**Published:** 2021-05-25

**Authors:** Rengasamy Balakrishnan, Shofiul Azam, Duk-Yeon Cho, In Su-Kim, Dong-Kug Choi

**Affiliations:** ^1^Department of Applied Life Science, Graduate School, BK21 Program, Konkuk University, Chungju 27478, Republic of Korea; ^2^Department of Biotechnology, College of Biomedical and Health Science, Research Institute of Inflammatory Disease (RID), Konkuk University, Chungju 27478, Republic of Korea

## Abstract

Parkinson's disease (PD) is the second-most common neurodegenerative chronic disease affecting both cognitive performance and motor functions in aged people. Yet despite the prevalence of this disease, the current therapeutic options for the management of PD can only alleviate motor symptoms. Research has explored novel substances for naturally derived antioxidant phytochemicals with potential therapeutic benefits for PD patients through their neuroprotective mechanism, targeting oxidative stress, neuroinflammation, abnormal protein accumulation, mitochondrial dysfunction, endoplasmic reticulum stress, neurotrophic factor deficit, and apoptosis. The aim of the present study is to perform a comprehensive evaluation of naturally derived antioxidant phytochemicals with neuroprotective or therapeutic activities in PD, focusing on their neuropharmacological mechanisms, including modulation of antioxidant and anti-inflammatory activity, growth factor induction, neurotransmitter activity, direct regulation of mitochondrial apoptotic machinery, prevention of protein aggregation via modulation of protein folding, modification of cell signaling pathways, enhanced systemic immunity, autophagy, and proteasome activity. In addition, we provide data showing the relationship between nuclear factor E2-related factor 2 (Nrf2) and PD is supported by studies demonstrating that antiparkinsonian phytochemicals can activate the Nrf2/antioxidant response element (ARE) signaling pathway and Nrf2-dependent protein expression, preventing cellular oxidative damage and PD. Furthermore, we explore several experimental models that evaluated the potential neuroprotective efficacy of antioxidant phytochemical derivatives for their inhibitory effects on oxidative stress and neuroinflammation in the brain. Finally, we highlight recent developments in the nanodelivery of antioxidant phytochemicals and its neuroprotective application against pathological conditions associated with oxidative stress. In conclusion, naturally derived antioxidant phytochemicals can be considered as future pharmaceutical drug candidates to potentially alleviate symptoms or slow the progression of PD. However, further well-designed clinical studies are required to evaluate the protective and therapeutic benefits of phytochemicals as promising drugs in the management of PD.

## 1. Introduction

Parkinson's disease (PD) is a common progressive chronic neurodegenerative movement disorder that increases with age. PD prevalence is 315 per 100 000 persons of all ages in the Western world; this prevalence is expected to double by the year 2030, increasing mortality, morbidity, and socioeconomic burden worldwide [1]. The clinical symptoms commonly associated with PD disorder include bradykinesia, resting tremor, postural instability, rigidity, depression, and anxiety [2]. The important hallmarks of PD are progressive loss or damage of dopaminergic neurons in the substantia nigra pars compacta (SNpc) and dopamine (DA) depletion in the striatum (ST), which is associated with the motor impairments of PD [3]. In addition to the neuropathological process affecting dopaminergic and nondopaminergic systems, other pathological processes are also seen in Alzheimer's disease (AD) affecting cholinergic dysfunction and serotonergic, glutamatergic, and noradrenergic pathways associated with dopaminergic neuronal death and/or DA system dysfunction. People with PD also experience nonmotor symptoms such as sleep disturbance, cognitive changes, autonomic dysfunction, altered mood, depression, fatigue, and pain [4, 5]. PD is described as a synucleinopathy, as accumulation of misfolded *α*-synuclein becomes a central feature of Lewy bodies, which are an important pathological hallmark of PD. Moreover, *α*-synuclein appears to be linked to both sporadic and familial forms of the disease and carries unique importance in the etiology of PD [6]. Interestingly, *α*-synuclein accumulation has been broadly linked to several neurotoxin pathways, including posttranslational modifications, neuroinflammation, oxidative stress, mitochondrial dysfunction, altered mitochondrial morphology, synaptic dysfunction, phospholipids, induced endoplasmic reticulum (ER) stress, and metal ions [7]. The age-related failure of antioxidant defense system and overproduction of ROS exacerbate oxidative stress in the brain; these events may play a role of misfolded *α*-synuclein initiating aging process in PD [8, 9].

Currently, levodopa (L-dopa) is the most effective therapy for the early-stage motor symptoms of PD, but it is not considered a cure for PD [10]. Bradykinesia and rigidity respond best, whereas tremor may be only slightly reduced. Problems with balance and other symptoms may not be alleviated at all. However, L-dopa is not effective in relieving neuronal loss, nonmotor symptoms, or Lewy pathology. Over time, patients require higher doses of L-dopa, which are associated with increased side effects such as dyskinesia [11]. Anticholinergic drugs may help control rigidity and tremor in approximately 50% of cases, and the antiviral agent amantadine also seems to diminish motor symptoms [3]. Deep brain stimulation (DBS) and DA-based medications are also used to treat various neurologic motor symptoms with disease progression [12]. Hence, it is critical to develop new therapeutic approaches to prevent neuronal loss and nonmotor symptoms and to prevent the accumulation of *α*-synuclein aggregation or Lewy pathology in the brain. Moreover, only symptomatic treatment options are available for PD; none slow or prevent progressive neuronal loss in the dopaminergic system [13, 14]. Herbal preparation and phytochemicals isolated from plant food have been proposed as “herbal medicine” for the treatment of PD [15]. Myriad phytochemicals from nature have been documented as potential molecules, drug leads, and phytochemical formulations in treating several inflammatory disorders [16, 17]. Likewise, extensive pharmacological reports have demonstrated the effectiveness of phytochemicals in treating dementia, depression, and neurodegenerative disorders (NDDs) [18]. Biologically active phytochemicals produced in plants are of clinical importance as primary and secondary metabolites for the antioxidant defense mechanism against various stress-related disorders and other pathogenic conditions [19]. The therapeutic and beneficial effects of these phytochemicals provide nutrition for normal living cells, fight disease-causing agents, strengthen the immune system, and act as antioxidants [20]. Plant products and their bioactive phytochemicals can efficiently scavenge oxygen free radicals and boost the cellular antioxidant defense system and related molecules, thereby protecting cells from oxidative damage [20, 21]. Several findings indicate that these antioxidant phytochemicals have confirmed neuritogenic potential, reconstructing synaptic connectivity by restoring the loss of neuronal processes [22–25]. In fact, various preclinical reports have described a number of natural pharmacological candidates that can coactivate the antioxidant defense system and neurotrophic factor-mediated cell survival systems [26–31], suggesting that these phytochemicals have therapeutic potential for the treatment of oxidative stress-mediated NDDs, especially PD.

Thus, therapeutic approaches targeting oxidative stress, *α*-synuclein accumulation, neuroinflammation, and mitochondrial dysfunction may hold great promise as a cure for PD. Numerous antioxidant phytochemicals have displayed potentially neuroprotective properties by targeting several mechanisms beyond those mentioned above. Phytochemicals are biologically active compounds that usually correspond to the secondary metabolites present in plants like alkaloids, flavonoids, and terpenoids [32]. Many epidemiological studies have suggested a proportional relationship between consumption of a diet rich in antioxidant phytochemicals and improved health outcomes, including reduced risk for AD, PD, and other NDDs [33–35]. Other epidemiological studies have associated the consumption of various food groups and beverages such as fruits, vegetables, tea, and coffee with reduced risk of development of PD [36, 37]. Recent research on the dietary intake of phenolic phytochemicals has been presented in several European countries, with results showing that the average intake is 820 mg/day in Spain, 1193 mg/day in France, and 1756.5 mg/day in Poland. The main dietary sources of the total polyphenols in Spain and France are fruits and nonalcoholic beverages (principally coffee and tea). In Spain, fruits accounted for 44% and nonalcoholic beverages for 23% of total polyphenol intake, whereas in France fruit accounted for only 17% and nonalcoholic beverages for 55%. Considered individually, the main source of total dietary polyphenols is food with 18% and 44% of contribution in Spain and France, respectively. In Spain, olives and olive oils are also important sources of polyphenols, accounting for 11% of the total polyphenol intake. Nonalcoholic beverages were the main food contributors to polyphenol intake in Poland and accounted for fully 67% of the total polyphenol intake due to high consumption of coffee and tea. The third main contributor to total polyphenol intake is chocolate, whereas fruits accounted for a lower percentage of intake [38–40].

**Figure 1:**
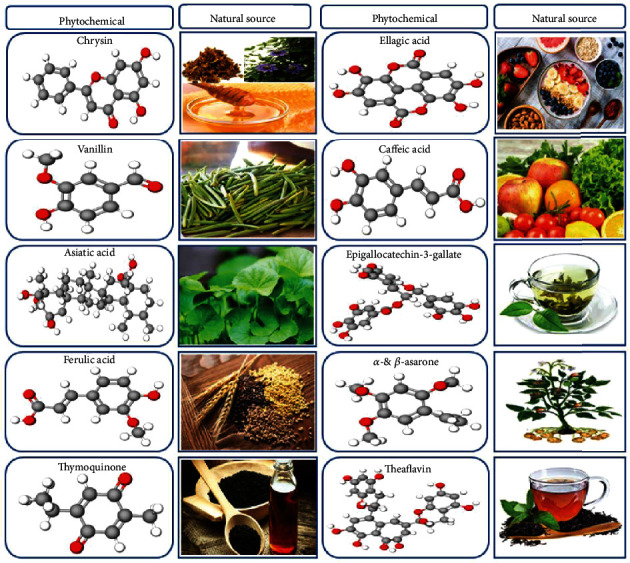
Structures of phytochemicals present in dietary sources (chrysin, vanillin, ferulic acid (FA), thymoquinone (TQ), ellagic acid (EA), caffeic acid (CA), epigallocatechin-3-gallate (EGCG), and theaflavin (TF)) and other plant-derived phytochemicals (asiatic acid (AA) and *α*- and *β*-asarone), belonging to different classes of phenolics and nonphenolics. These phytochemicals have demonstrated several mechanisms of action by which they protect the brain from neurodegeneration. The structures were regenerated from http://molview.org/. Here, the different colors of different atoms in the structures represent specific molecules, where grey = carbon, white = hydrogen, and red = oxygen.

In the present study, we describe the phytochemicals present in dietary sources, using chrysin, vanillin, ferulic acid (FA), thymoquinone (TQ), ellagic acid (EA), caffeic acid (CA), epigallocatechin-3-gallate (EGCG), theaflavin (TF), and other plant-derived antioxidant phytochemicals (asiatic acid (AA) and *α*- and *β*-asarone) as examples and discuss their beneficial neuroprotective effects and relevance to potential treatment strategies of PD. Importantly, phytochemicals have thus far been investigated primarily in both cellular and rodent experimental studies for their potential benefits in brain metabolism; these studies have provided some encouraging results indicating antioxidant, anti-inflammatory, and cognitive enhancing effects of these phytochemicals coupled with a wide range of tolerability [41, 42]. Additionally, phytochemicals also have been confirmed to reduce mitochondrial dysfunction and inhibit formation of *α*-synuclein accumulation-induced oxidative stress and inflammatory responses [43, 44]. Several studies have also provided evidence that the antioxidant activity of some phytochemicals can activate nuclear factor E2-related factor 2 (Nrf2)/antioxidant response element (ARE) signaling pathways. Furthermore, phytochemicals contribute to the activation of the phosphatidylinositol 3-kinase/protein kinase B (PI3K/Akt) and extracellular signal-regulated kinase (ERK) pathways and inhibit nuclear factor kappa B (NF-*κ*B) pathways [22, 45]. Similarly, a number of studies have suggested that phytochemicals confer neuroprotection in experimental parkinsonism by reducing oxidative stress and mitochondrial dysfunction and fostering degradation of *α*-synuclein toxic species through activation of autophagy [46–48]. This study provides information about the neuroprotective properties and mechanisms of action of recently discovered naturally derived phytochemicals that target oxidative stress and neurodegeneration through cellular- and molecular-level changes in the progression of PD. In addition, we explore Nrf2/ARE and autophagy signaling-related pharmacological mechanisms. Moreover, we highlight some potentially neuroprotective active derivatives of antioxidant phytochemicals and phytochemical-based nanodelivery systems that fight pathological conditions associated with aging-related oxidative stress. The sources and chemical structures of phytochemicals are presented in Figure 1.

## 2. Role of Oxidative Stress in PD

**Figure 2:**
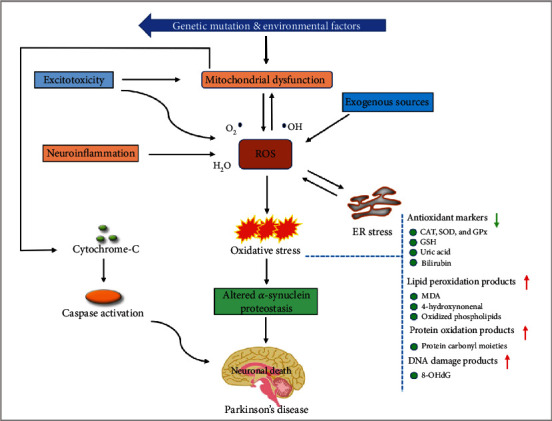
Oxidative stress and its implications in the pathogenesis of neurodegeneration in PD. When the production of ROS generation overwhelms intracellular antioxidant defenses, brain cells are exposed to oxidative stress, which may lead to mitochondrial dysfunction and further ROS production. Oxidative stress impairs the protein degradation system and hinders the clearance and results in the subsequent deposition of misfolded protein, which in turn results in lipid peroxidation, protein oxidation, and DNA damage, leading to neuronal death. These events constitute the pathological basis of PD. CAT: catalase; SOD: superoxide dismutase; GPx: glutathione peroxidase; GSH: glutathione; MDA: malondialdehyde; 8-OHdG: 8-hydroxydeoxyguanosine.

Oxygen is a crucial molecule that may produce free radicals during metabolic reactions. These free radicals constitute an important fundamental molecule in any biochemical and/or biological reduction reaction [49]. However, these free radicals are highly unstable and easily cross biomolecules like nucleic acid, lipids, cellular membranes, and proteins [50]. In addition, recent investigations have observed novel developments in the biology of free radicals and their impact on brain health and the development of various pathological conditions, since the brain uses a disproportionately large volume of oxygen compared to other organs, making it more vulnerable to free radical attacks [51, 52]. Free radicals like reactive nitrogen species (RNS) and reactive oxygen species (ROS) are prime stimulators of oxidative stress and imbalance in the antioxidant capability of cells [53]. ROS, such as hydrogen peroxide (H_2_O_2_), are key precursors of superoxide radicals (O^·^−2) produced in the brain mitochondria that restrict mitochondrial movement and damage DNA, leading to impaired brain function and NDDs [54]. Considerable evidence from recent *in vivo* experiments has confirmed that excessive production of ROS contributes significantly to neuronal cell death and altered brain function resulting from DA metabolism, low levels of glutathione (GSH), and high levels of calcium and iron in the substantia nigra (SN) [55, 56]. Moreover, the brain contains a high level of membrane polyunsaturated fatty acids, which, under oxidative stress conditions, leads to higher lipid peroxidation and the generation of neurotoxic products. These deficient enzymatic antioxidant systems and increased oxidative stress markers are common pathological hallmarks of PD [57]. ROS contributions to PD pathogenesis have been supported by extensive evidence (Figure 2).

Several studies have previously reported that PD tissues show an oxidant status that is broadly associated with both DA autooxidation and mitochondrial complex I dysfunction [58]. In addition, preclinical and clinical findings have revealed that brain and cerebrospinal fluid samples from PD patients contain significantly higher levels of oxidized coenzyme Q10 and the nuclear DNA oxidation biomarker 8-OHdG, establishing the existence of oxidative damage to DNA and mitochondria in PD patients [59]. Moreover, reduced mitochondrial complex I catalytic activity, downregulated mitochondrial biogenesis, and reduced mitochondrial DNA levels in the prefrontal cortex were observed contributing to excessive ROS production and oxidative stress in PD brains [60]. Recent findings have identified excess production of ROS and diminished mitochondrial complex I activity in SH-SY5Y neuroblastoma cells [61]. Similarly, one model using 6-hydroxydopamine- (6-OHDA-) induced rats found increased levels of 8-OHdG in the urine, serum, and SN of rats when compared with control groups; this finding was consistent with research involving human tissue [62]. In addition, earlier studies have demonstrated that increased ROS production causes significant and simultaneous dysregulation of several signaling pathways, such as RAS-MEK-ERK1/2 [63], PI3K/AKT/GSK3*β* [64], Keap1-Nrf2-ARE [65], NF-*κ*B [66–68], and JAK/STAT [69].

## 3. Role of Autophagy in PD

Autophagy is the key physiological cellular catabolic process in response to cellular starvation and the degradation of damaged organelles [70]. Autophagy describes the process by which diverse cellular mechanisms encompassing nucleic acids, whole organelles, lipids, proteins, sugars, and cytoplasmic compartments are sequestered into a double-membrane budding vacuole called a phagophore, which later matures to seal in a vesicle called an autophagosome [71, 72]. Damaged or dysregulated autophagy has been linked with several pathological processes, including inflammation, cancer, lipid metabolism, and NDDs [73–76]. In fact, autophagy helps clear damaged organelles, protein aggregates, and lipid droplets, which constitute unwanted and often toxic cargo that may lead to cellular dysfunction. Importantly, damaged or dysregulated autophagy has been recognized as a critical pathogenic process, particularly in PD and other NDDs [73, 77].

In PD, the role of autophagy is significant in neuronal quality control and brain maintenance. Recent confirmations have demonstrated that mice lacking key autophagy genes like AuTophaGy ATG5 and ATG7 show spontaneous neurodegeneration, accumulation of protein aggregates, and motor neuron dysfunction [78, 79]. 6-OHDA, 1-methyl-4-phenylpyridinium (MPP^+^), rotenone, and 1-methyl-4-phenyl-1,2,3,6-tetrahydropyridine (MPTP)—a neurotoxin associated with mitochondrial dysfunction, ATP depletion, and accumulation of ROS such as H_2_O_2_, hydroxyl radicals, and superoxide—are commonly used in experimental models of PD in both *in vitro* and *in vivo* research [80, 81]. Changes in autophagy status have been reported in 6-OHDA-induced PD models [82, 83]. Recently, it was reported that a mild increase in ROS levels could activate mucolipin 1, a key calcium-conducting channel located on the lysosome membrane, to initiate calcineurin-dependent activation of transcription factor EB, which is identified as a master regulator of the autophagy–lysosome pathway (ALP) [84]. In turn, the transcription factor EB-mediated induction of autophagy promotes clearance of damaged mitochondria and removal of excess ROS [73]. Excessive ROS levels may cause lysosomal dysfunction and autophagic failure and lead to oxidative stress or cell death; however, oxidative stress generally mediates autophagic pathway activation [85]. Moreover, the activation levels and induction time of autophagy also play critical roles in the survival or death of cells [86].

As mentioned previously, DJ-1 has been confirmed to regulate basal autophagy and mitophagy in a manner similar to Parkin and PINK1 [87, 88]. Recently, it has been demonstrated that DJ-1 loss-of-function mutations were first identified as generating oxidative stress in mice and *Drosophila* exposed to the toxicity of rotenone, paraquat (PQ), and MPTP [89, 90]. Interestingly, DJ-1 overexpression has been shown to protect against oxidative insults. In dopaminergic cell lines, the overexpression of DJ-1 was able to protect dopaminergic cells through reduced levels of protein oxidation, ROS, and cell death in H_2_O_2_- and 6-OHDA-exposed wild-type, but not mutant cells [91, 92]. In animal models, DJ-1 overexpression has been shown to protect against dopaminergic neuronal degeneration in mice exposed to MPTP and rats exposed to 6-OHDA in wild-type, but not mutant murine models [91, 93]. Similarly, novel drugs activating DJ-1 protected against rotenone- and 6-OHDA-induced degeneration of SN dopaminergic neurons in murine PD models [94]. The findings of the previous studies suggested that these neuroprotective effects of DJ-1 may diminish ROS production in response to local L-calcium channels' pacemaking activity in dopaminergic neurons through regulation of mitochondrial functions [95]. Overall, the excess ROS and RNS are generated when autophagy and mitophagy are impaired. Consequently, in the present study, we explore evidence of the autophagy-based neuroprotective effects of some natural phytochemicals in experimental PD.

## 4. Oxidative Stress and Antioxidant Defense Mechanisms

Nrf2 is a ubiquitous transcription factor that binds to the ARE in the maintenance of intracellular homeostasis and protects cells from harmful chemical agents by inducing the detoxifying agent and responding to oxidative stress. Under normal conditions, three known ubiquitin ligase systems, E3 ubiquitin ligase Hrd1, glycogen synthase kinase (GSK3*β*), and Kelch-like ECH-associated protein 1 (Keap1), contribute extensively to the degradation of Nrf2 [96]. The electrophilic nature of these three activator genes enables the modification of thiol group (-SH) groups in Keap1 via oxidation, alkylation, and reduction processes [97]. Under oxidative stress condition, the Nrf2-ubiquitin ligase system's interaction is disturbed, and newly synthesized Nrf2 is transported into the nucleus, where it binds to the ARE region of specific target genes [98]. These targets are typically considered to be antioxidant genes; however, in recent decades, several scientific findings have demonstrated the extensive involvement of Nrf2 in the regulation of various physiological functions through its antioxidant, anti-inflammatory, autophagic, detoxifying, and proteasomal actions [99, 100]. Some of the physiological functions are directly or indirectly linked with PD pathogenesis. Previous research has examined the Nrf2 signaling-mediated antioxidant protection of neurons in numerous PD models, finding that Nrf2 directly regulates the activities of catalase (CAT), sulfiredoxin-1 (SRXN1), NAD(P) H:quinone oxidoreductase-1 (NQO1), superoxide dismutase 2 (SOD2), heme oxygenase-1 (HO-1), peroxiredoxins 3 and 5 (PRDX3 and PRDX5), and enzymes involved in GSH metabolism [101–103]. Genetic variation in the Nrf2 gene has been associated with PD progression [98]. Recently, microchip analysis revealed that Nrf2-dependent signaling cascades play a vital role during PD; this role was well illustrated in various types of tissues of patients with PD. The expression of 31 genes that contain ARE sequences in the promoter decreased significantly, while the expression of Nrf2 increased in all of the tissue samples of PD patients [104].

Recently, several *in vitro* and *in vivo* findings provided evidence for a possible protective role of the Nrf2-ARE pathway in PD. For instance, apomorphine, which acts a dopamine D(1)/D(2) receptor agonist, activates the Nrf2-ARE signaling pathway to protect against ROS-mediated damage, thereby bolstering the cellular defense system. Pretreatment of SH-SY5Y cells with apomorphine stimulated the translocation of Nrf2 into the nucleus and the transactivation of the ARE. The expression of HO-1 was induced by apomorphine in a dose-dependent manner. Moreover, the authors found that the activation of the ARE and the induction of HO-1 mRNA caused by apomorphine were suppressed in the presence of intracellular ROS in 6-OHDA-induced SH-SY5Y cells [105]. Transplantation of astroglia cells overexpressing Nrf2 into mouse brain showed a significant decline in vulnerability to 6-OHDA-mediated neurotoxicity [106]. Similarly, astrocytic Nrf2 overexpression diminished MPTP-mediated toxicity in mice, demonstrating that astrocytic modulation of the Nrf2-ARE pathway indicates decreased or attenuated neuronal death in PD [107]. Deprenyl, a drug used in the treatment of PD, was shown to stimulate Nrf2 activity as part of its cytoprotective mechanism of action [108]. Another study reported that flavonoid luteolin effectively protects against the mitochondrial toxin MPP^+^ by activation of the Nrf2 pathway in cultured rat neuronal PC12 and glial C6 cells [109]. In a more recent study, the knockdown of Nrf2 nullified this protective effect, showing that luteolin-triggered protection is mediated solely through Nrf2. Similarly, the Nrf2 transcriptional activator tBHQ protected against neurotoxin deltamethrin-induced PC12 in a cellular PD model [110]. In a very recent study using an MPTP-induced PD model, uric acid increased Nrf2-responsive genes, including g-glutamate-cysteine ligase catalytic subunit (g-GCLC), HO-1, NQO1, and mRNA, and protein expressions of Nrf2 [111]. Sulforaphane, a natural phytochemical, significantly attenuated cell damage and reversed the reduction of Nrf2, HO-1, and NQO1 expression induced by MPP^+^ neurotoxicity; these protective effects are due in part to the activation of the Nrf2-ARE signaling pathway [112]. A similar protective effect against the decreased levels Nrf2 and Keap1 caused by 6-OHDA has been reported in rats treated with puerarin; the puerarin decreased lipid peroxidation and oxidative stress in the SN, significantly increased brain-derived neurotrophic factor (BDNF) expression, and activated the Nrf2/ARE signaling pathway [113]. Another study reported that dietary bioflavonoid pinostrobin significantly enhanced Nrf2 expression and nuclear accumulation, improved ARE promoter activity, and upregulated expression of HO-1. Interestingly, authors then found that pinostrobin promoted phosphorylation of PI3K/AKT and ERK, and pharmacological inhibition of PI3K/AKT or ERK signaling diminished pinostrobin-induced Nrf2/ARE activation and neuroprotective actions against MPTP/MPP^+^-induced neurotoxicity in PD models [114]. Together, these findings strongly indicate that treatment of PD via activation of the Nrf2-ARE signaling pathway may be possible. Moreover, these studies demonstrate the protective effects of naturally derived antioxidant phytochemicals on Nrf2-ARE-induced antioxidant protection in experimental models of PD.

## 5. Structure-Activity Relationship between Phytochemicals and Neuroprotection in PD

Recent reports have shown that polyphenols, terpenoids, flavonoids, ascorbic acid, alpha-tocopherol, catechins, and beta-carotene may offer a potential neuroprotective effect and play a role in alleviating the progression of PD [39, 115]. Polyphenolic compounds have been found in a variety of edible and nonedible plants; to date, approximately 8000 different flavonoids have been identified in different organs of plant tissues [116, 117]. ROS generation and RNS in mitochondria and nicotinamide adenine dinucleotide phosphate (NADPH) oxidase can trigger oxidative damage harmful to cells and decrease antioxidant defense, resulting in age-associated NDDs such as PD [118, 119]. Moreover, free radical-mediated oxidation of nucleic acids, lipid oxidation, and oxidation of proteins can damage cell membranes and trigger protein crosslinking [120]. The health benefits of flavonoids and terpenoids are attributed to their antioxidant and chelating abilities. Through their capacity for oxidation, terpenoids and flavonoids have confirmed unique neuroprotective effects [39, 121]. Flavonoids give a proton to form a phenoxyl radical and a singlet oxygen and scavenge superoxide as well as hydroxyl and peroxyl radicals by the release of an additional proton. Indeed, a complex structure was formed by the diol group with copper, various transition metal ions, and ferric iron, which plays a crucial role in preventing the production of ROS [122]. Furthermore, recent research revealed that flavonoids, terpenoids, and nonflavonoids chelate copper and iron ions and reduce the generation of free radicals [123].

The structure required for neuroprotective activity is not always linked with antioxidant properties [124]. Previous studies have demonstrated that EGCG, TF, and EA and its derivatives have good free-radical-scavenging effects and relatively high hydrophobic properties; thus, these compounds showed much higher antioxidant activity [125, 126]. These results suggest the relationship of independent antioxidant mechanisms in their neuroprotective activity. Furthermore, glutathione reductase (GR), GPx, CAT, SOD, and glutathione S-transferases (GSTs) are polyphenols that upregulate ROS-scavenging enzymes [127]. Polyphenols may enhance the levels of these antioxidants by activation of cellular signaling pathways [128]. Phenolic acids of FA, EA, and CA have 4-hydroxy and electron-donating 3-methoxy groups on the benzene ring and can scavenge hydroxyl, peroxynitrite, and superoxide and terminate radical chain reactions. These phenolic acids effectively bind to the lipid bilayer and prevent lipid peroxidation with an adjacent unsaturated carbon–carbon double bond [129, 130]. There is evidence that phenolic acids and their ethyl ester derivatives can upregulate protective genes, such as heat shock protein-70 (HSP-70), HO-1, and ERK1/2, which are beneficial therapeutic and preventive agents in NDDs, particularly in PD [129, 131].

The chemical properties of chrysin, due to a lack of B- and C-ring oxygenation, are linked with several pharmacological properties that range from antioxidant to anti-inflammatory effects [132]. Differences in the chemical structure of flavones have been shown to enhance the activity of antioxidant enzymes and to provide an inhibitory effect on the proinflammatory mediator of COX-2 expression [133]. In addition, xanthine oxidase inhibitory assay has shown that chrysin significantly suppresses xanthine oxidase production of ROS [134, 135], and amentoflavone and blueberry potentially inhibit iNOS expression [136]. Interestingly, in rodent models, 10–100 *μ*M concentrations are required to display the potential antioxidant and anti-inflammatory activities of polyphenols [134, 135].

## 6. Neuroprotective Roles of Phytochemicals: Antioxidant, Anti-Inflammatory, and Other Signaling Pathways

### 6.1. Neuroprotective Mechanisms of Chrysin in PD

Chrysin is a natural polyphenolic compound known as a flavonoid; flavonoids are ubiquitous in vegetables, fruits, mushrooms, blue passion flowers, plants, and especially in honey [137]. Numerous epidemiological, cellular, and animal studies have substantiated the potential health benefits of flavonoids. The main sources of flavonoids in Westernized countries—specifically Australia, the United States, and many European nations—are tea, fruit or vegetable juices, blue passion flowers, and wine, with the estimated total intake of flavonoids ranging from 200 mg/day in Australia [138] to 500 mg/day in parts of Europe where tea consumption is high [139]. In countries such as Spain, Poland, Mexico, and Greece, where diets are rich in citrus fruits and wine, dietary flavanones can range from 30 mg/day (Greece) to 170 mg/day (Poland) [139–142]. Similarly, in Asian countries (China, Japan, and Korea), dietary flavanone intake ranged from 36 mg/day (Korea) to 5 mg/day (China) [143–146]. Chrysin has been explored for its neuroprotective effects, which are attributable to its antioxidant, anti-inflammatory, and other pharmacological properties. The various therapeutic activities of chrysin depend on its bioavailability and attainable concentrations in cells and target tissues in rodents. Following administration of 400 mg of chrysin to human subjects, bioavailability was found to be extremely low due to rapid metabolism, poor intestinal absorption, and rapid excretion [147]. Scientific evidence has established that chrysin is found conjugated approximately 99% in the plasma protein binding [147]. However, it was reported that chrysin is considered to have very low oral bioavailability and a distribution volume of approximately 0.003%–0.02% [147]. Furthermore, a recent investigation demonstrated that chrysin can cross biological membranes easily; however, extensive sulfation and glucuronidation in the intestinal cells limit its absorption [148]. Although chrysin is considered safe for human consumption at a daily dose of 500 mg to 3 g without any toxic effects, higher doses may induce undesirable side effects [149]. Chrysin is especially important for polyphenols targeting the brain; penetration of the blood-brain barrier (BBB) is crucial for their therapeutic benefits [150].

The neuroprotective activity of chrysin shows that increases in DA levels in both *in vitro* and *in vivo* experiments are inversely associated with dopaminergic neuronal loss (Table 1) [151, 152]. It was found that chrysin enhanced DA levels by acting as an Monoamine oxidase B (MAO-B) inhibitor in MPP^+^- and MPTP-treated CGN cells and mouse models of PD [152]. In this way, chrysin treatment can increase DA levels and subsequently suppress DA metabolism in the ST through the inhibition of MAO-B [152]. Several neurotoxin-induced *in vivo* experiments also demonstrated that chrysin increased DA, 3,4-dihydroxyphenylacetic acid (DOPAC), and homovanillic acid (HVA) levels [153, 154]. Moreover, chrysin administration very significantly attenuated the cognitive dysfunction and motor impairment in these animals as evaluated by passive avoidance, rotational behavior, and Barnes maze tests [154], as well as beam walk and horizontal and vertical grid tests [153]. Therefore, chrysin presents as a potential disease-modifying agent that may also prove beneficial for symptom relief in PD.

Several preclinical studies have revealed that chrysin provides antioxidant effects by reducing oxidative stress and modulating or boosting cellular antioxidant enzyme levels, thus decreasing the lipid peroxidation triggered by numerous oxidative insults [155–157]. In aged mice, chrysin administration significantly blocked the elevation of ROS levels and boosted antioxidant enzyme activities [158]. In the MPTP-induced PD mouse model, chrysin pretreatment diminished oxidative stress as evaluated by lipid peroxidation levels; the pretreatment also inhibited reduction of SOD activity and GSH content in the SN region [153]. In agreement with these results, chrysin administration inhibited the increase of NO and NADPH oxidase activity and prevented the reduction of GSH in the ST of 6-OHDA-treated mice [154]. Additionally, chrysin treatment attenuated 6-OHDA-induced decreases in the levels of Na^+^ and K^+^-ATPase in the ST of mice. Na^+^ and K^+^-ATPase play a central role in maintaining ionic gradients and neuronal excitability and are more susceptible to oxidative damage [159].

RNS generated by iNOS may be significantly associated with oxidative stress in PD, and excessive production of NO can lead to loss of dopaminergic neurons and impaired motor functions [160]. NADPH oxidase activation can stimulate ROS and plays a vital role in dopaminergic neurodegeneration [161]. In this regard, pretreatment of PC12 cells with chrysin may reduce the intracellular NO level, decrease iNOS expression, and subsequently inhibit NF-*κ*B phosphorylation and decrease its transcriptional activity induced by 6-OHDA neurotoxicity [162]. In particular, chrysin administration significantly enhanced the anti-inflammatory markers interleukin-4 (IL-4) and interleukin-10 (IL-10), diminished expression of tumor necrosis factor-alpha (TNF-*α*), interleukin-1*β* (IL-1*β*), and interleukin-6 (IL-6), and inhibited myeloperoxidase (MPO), COX-2, and iNOS expression, which are highly implicated in anti-inflammatory responses [163]. In a cerebral ischemic mouse model, chrysin inhibited neuroinflammation by reducing the level of IL-1*β* and TNF-*α* expression, prevented cognitive deficits, and downregulated the activation of NF-*κ*B [164]. In addition to inhibiting NF-*κ*B activation and downregulating gene-related inflammation, chrysin also interacts with iNOS enzymes at a molecular level and COX-2 and decreases levels of arachidonic acid-induced prostaglandins, thromboxanes, and leukotrienes in the SN and ST of 6-OHDA- and MPTP-treated PD mice [151, 153]. Hence, one may conclude that chrysin's protective effects may carry promising anti-inflammatory properties in PD that are at least partially mediated by NF-*κ*B inhibition.

In an experimental model using MPTP, chrysin administered to mice was demonstrated to attenuate dopaminergic neuronal loss in the SN region [152]. Consequently, immunohistochemical analysis revealed that oral administration of chrysin significantly restored the loss of TH-positive cells in the ST and protected nigrostriatal morphology against 6-OHDA-induced mouse models of PD [151]. A very recent study showed that oral administration of chrysin improved locomotor activity and protected against dopaminergic neurodegeneration in MPTP-injected PD mouse models [153]. Chrysin played a neuroprotective role at a molecular level, at least in part through the suppression of proapoptotic proteins such as caspase-3, caspase-9, and Bax, and enhanced antiapoptotic protein Bcl-2 expression against MPP^+^ neurotoxicity [152]. Moreover, chrysin treatment provided neuroprotectivity by regulating or restoring BDNF and glial cell-derived neurotrophic factor (GDNF) levels in the ST region in 6-OHDA- and MPTP-induced PD mouse models [151, 153]. Importantly, PD animal models showed that neurotrophic factors such as BDNF and GDNF could partially inhibit neurodegeneration as reported by previous studies [165, 166]. Hence, the multiple protective roles of chrysin in PD, both neuroprotective and symptom-relieving, are of great value and may open new horizons for novel therapeutic management of PD; further clinical studies are needed.

### 6.2. Neuroprotective Mechanisms of Vanillin in PD

Vanillin is a phenolic aldehyde compound used as an important flavoring agent worldwide. It is found in abundance in plant species and is often used in the food production, beverage, pharmaceutical, perfume, and cosmetic industries [191]. Currently, approximately 50% of worldwide synthetic vanillin production is used as an intermediate in both food and nonfood applications and in pharmaceutical industries for the production of herbicides, antifoaming agents, or drugs such as papaverine, L-dopa, L-methyldopa, and antimicrobial agents [191]. Acceptable daily intake of vanillin is consumed in the form of food and beverage worldwide, and its ubiquity suggests that nearly every human consumes vanillin-containing products. A daily intake of vanillin 10 mg/kg has been approved by the Food and Agriculture Organization of the United Nations (FAO)/WHO and European Union. For a person weighing 70 kg, the daily recommended intake is 700 mg of vanillin, which amounts to a minimum of 700 g of chocolate or 700 g of ice cream [192].

Vanillin has been studied extensively for its pharmacological properties, which are attributable to its structure and main bioactive metabolites, including vanillyl alcohol and vanillic acid; vanillin's bioactive properties include antioxidant, anti-inflammatory, and neuroprotective abilities [193]. Vanillin can easily penetrate the BBB and demonstrates significant brain-neuroprotective potential by enhancing the activities of antioxidant enzymes and reducing the levels of lipid peroxidation and NO production [194, 195]. This polyphenolic flavoring agent can scavenge the O^·^−2 and ^·^OH intermediates implicated in biological membrane damage [196]. It alleviates renal oxidative stress by decreasing lipid peroxidation levels, increasing levels of enzymatic (SOD, CAT, GPx, and GSH) and nonenzymatic antioxidants (vitamin C and nonprotein thiol), and protecting against DNA damage and histopathological changes in maneb-induced mice [197]. Another study demonstrated the neuroprotective properties of vanillin in a rotenone-induced PD model. Administration of vanillin in SH-SY5Y cells inhibited rotenone-induced ROS generation, mitochondrial dysfunction, and caspase activation and reduced the expression of signaling molecules [198].

A previous study elucidated the relationship between striatal DA depletion and motor impairments [199]. Furthermore, intraperitoneal injection of rotenone displayed a significant diminishment in locomotion activity, akinetic movement, and cataleptic ability [200]. Oxidative stress-mediated degeneration of neurons was further exacerbated by high levels of DA metabolism, greater prevalence of glial cells, and low levels of antioxidant enzyme activity in the SN and ST regions [201]. The successful antioxidant actions of vanillin are due to its structure by reducing the levels of lipid peroxidation and enhancing the antioxidant enzyme activities [168, 202]. A recent study determined that vanillin treatment increases the striatal DA and its metabolites, subsequently improving behavioral function. It has also been reported to prevent Cyto-C release, diminish Bax expression, inhibit caspase activation, and enhance Bcl-2 expression in rotenone-induced rat models of PD [168]. Vanillin has also exhibited improvement in chronic stress-induced rat models by reducing the depressive-like motor symptoms via elevation of serotonin and DA levels in brain tissue [203].

Other study findings recently revealed that mice that inhaled vanillin had a decreased pain response to the hot plate test; no significant differences were observed between the inhaled vanillin group and the control group in the tail suspension, Y-maze, forced swimming, open field, and aggression tests. These results suggested that inhaled vanillin has potential antinociceptive properties similar to other routes of administration [204]. In LPS-lesioned PD models, oral administration of vanillin reduced IL-1*β*, IL-6, and TNF-*α* expression through inhibition of the p38-MAPK signaling pathway, suppressing activation of NF-*κ*B and inflammatory genes like iNOS that produce NO and COX-2. Consequently, LPS-lesioned rats experienced increased degeneration of dopaminergic neurons and decreased motor function, and microglial activation triggered in the SN and ST was restored or improved significantly following vanillin treatment [167]. Similar findings also confirmed that vanillin reduced the inflammatory mediators of iNOS and COX-2, as well as the mRNA expression levels of proinflammatory cytokines. In addition, vanillin effectively inhibited the phosphorylation of MAPK signaling and NF-*κ*B activation in LPS-lesioned microglia cells [205]. Together, these data suggest the neuroprotective and anti-inflammatory role of vanillin in protecting dopaminergic neurons and improving behavioral function by inhibiting oxidative stress, inflammation, and apoptosis; thus, it is possible that vanillin may act as a natural therapeutic drug for PD.

### 6.3. Neuroprotective Mechanisms of Asiatic Acid in PD

Asiatic acid (AA), a naturally pentacyclic triterpenoid, shows promise as neuroprotective drug candidate due to its several pharmacological properties. Interestingly, a number of bioactive components of AA were found to have therapeutic potential in curing various diseases [206, 207]. AA exhibited the ability to modulate several enzymes, growth factors, receptors, transcription factors, apoptotic proteins, and cell signaling molecules [208, 209]. In experimental studies, AA has displayed a wide range of biological activities, including antioxidant, hepatoprotective, antidiabetic, anticancer, anti-inflammatory, and neuroprotective properties [210–213]. Recently, AA tromethamine-loaded solid lipid nanoparticles have been shown to prevent proteolytic degradation and to facilitate sustained release of the drug, thereby improving its bioavailability [214]. Another study injected formulations containing glutathione-conjugated BSA nanoparticles of AA intravenously into rats at a dose of 75 mg/kg; after 5 hours, the nanoformulation displayed ten times greater bioavailability in the brain than AA alone [215].

AA provides neuroprotection by maintaining the stability of the BBB and by protecting mitochondrial functions. A recent *in vitro* study showed that AA administration reduced intracellular mitochondrial ROS production and altered MMPs to regulate mitochondrial function, subsequently decreasing NLRP3 inflammasome expression in microglia cells [216]. In addition, AA treatment directly improved SH-SY5Y cell viability and maintained mitochondrial function in an MPP^+^-induced PD model [216]. Furthermore, administration of AA attenuated ROS overproduction, mitochondrial dysfunction, and apoptotic expressions of proapoptotic and antiapoptotic indices in a rotenone-induced SH-SY5Y PD model [217]. A similar study reported that AA pretreatment protected cell viability, inhibited the upregulation of voltage-dependent anion channel mRNA and protein expression levels, and prevented MMP damage following exposure to H_2_O_2_ [218]. Further recent *in vitro* and *in vivo* experiments also observed that the administration of AA significantly decreased apoptotic cell death, lessened mitochondrial ROS production, stabilized MMPs, and promoted the expression of PGC1*α* and Sirt1 to mitigate toxicity induced by glutamate in a dose-dependent manner. In the *in vivo* model, oral administration of AA significantly attenuated cognitive deficits, decreased lipid peroxidation level, and restored GSH content and SOD activity in the hippocampus and cortex; subsequently, AA effectively attenuated glutamate-induced neuronal damage of the pyramidal layer in the CA1 and CA3 regions [219].

Moreover, the anti-inflammatory activity of AA significantly decreases the level of MDA, NO, and inflammatory mediators iNOS and COX-2 and has also been shown to inhibit NF-*κ*B activation in paw edema by increasing the antioxidant enzyme levels of CAT, GPx, and SOD in *λ*-carrageenan-induced mice [220]. In the MPTP/p-induced PD mouse model, AA administered was shown to improve behavioral function, enhance DA levels, and increase expressions of neurotrophic factors and tyrosine kinase receptors (TrKB). Moreover, AA significantly inhibited phosphorylation of P38, JNK, and ERK protein expression and significantly increased phosphorylated PI3K, Akt, GSK-3*β*, and mTOR activation [170]. Indeed, experimental studies showed that AA treatments increased striatal DA levels and upregulated striatal TH, TLR4, BDNF, and GFAP expression, subsequently decreasing striatal upregulation of *α*-synuclein, apoptotic markers, and Bcl-2 expression in MPTP-induced PD-like neurotoxicity in mice. In addition, posttreatment of AA significantly suppressed NF-*κ*B activation [169]. Interestingly, recent studies have also found that AA prevented decreases in Nrf2 expression, counteracted the downregulation of neurogenesis within the hippocampus, and mitigated memory deficits produced by 5-fluorouracil through inhibiting oxidative stress and boosting the antioxidant defense system [221]. These novel findings suggest that AA may be developed as an agent for PD prevention or therapy.

### 6.4. Neuroprotective Mechanisms of Ferulic Acid in PD

Ferulic acid (FA) is a natural phenolic phytochemical commonly found in apples, oranges, peanuts, wheat, rice, barley, coffee, and many other dietary sources [222]. Investigations have confirmed the beneficial effect of FA-rich foods and drinks. Recent reports revealed that consumption of vegetables, fruits, and cereals is beneficial in the prevention of diabetes, cancer, obesity, cardiovascular disease, AD, and PD [129, 223, 224]. In Japan, FA has been approved as a food additive and is used as a natural antioxidant in foods, beverages, and cosmetics. In the United States and most European countries, numerous medical essences and natural extracts of herbs, coffee, vanilla beans, spices, and other botanicals are selected for their high content of FA and are added to foods as an FDA-approved antioxidant concoction [225]. Accurate nutritional surveys about FA intake are lacking, but consumption of FA from food sources can be estimated at a daily intake of approximately 200–1000 mg per person [222, 226, 227].

In recent years, scientific findings have reported the health benefits of FA, which exhibits low toxicity and possesses several pharmacological properties including immunomodulatory, antioxidant, anti-inflammatory, antiapoptotic, anticancer, antidiabetic, and neuroprotective functions. FA achieves these functions through inhibition of lipid peroxidation and ROS generation by its phenolic hydroxyl group. Moreover, FA has been reported to downregulate the expression of enzymes that promote inflammation [129, 222]. The physiological benefits of FA depend on its bioavailability for absorption and consequent interaction with target tissues. Recent preclinical investigations estimated that for subjects who consumed phenolic acid-containing drinks, vegetables, and fruits in the daily diet, total daily intake of polyphenols equaled about 1 g [227]. In animal models, FA has shown greater bioavailability compared to other various phenolic ingredients [222, 228].

Based on its structural similarity to another phenolic compound, salicylic acid, that can enter the brain, it is speculated that FA can easily penetrate the BBB. It has also been reported to be a successful neuroprotective agent [229]. Recent *in vitro* and *in vivo* findings have demonstrated that FA exhibited increased levels of protective HO-1 activity in SH-SY5Y cells, upregulated the levels of CAT, SOD, GPx, and GSH, and reduced the lipid peroxidation level in MPTP-injected PD mouse models, which confirms FA's potential antioxidant effects in the prevention of PD [230]. In another study, FA led to upregulation of antioxidant enzyme status through induced HO-1 gene expression, enhanced ARE promoter activity, promoted ERK1/2 phosphorylation, and Nrf2 translocation in PC12 cells exposed to lead acetate [231]. Moreover, oxidative stress stimulated by 6-OHDA and rotenone was alleviated effectively by FA treatment via its ability to scavenge radicals generated by increased lipid peroxidation, decreased antioxidant GSH content, and mitochondrial oxygen radical production, thus substantiating FA's preventive effect against oxidative damage in PD [171, 232].

Anti-inflammation has been proposed as one of the main mechanisms underlying FA-induced neuroprotection in PD, and several studies have shown that FA can potentially inhibit neuroinflammation and neuronal apoptosis in NDDs. Furthermore, histological findings revealed that FA administration suppressed microglial cell activation and the Bax/Bcl-2 ratio, reflecting a reduction in inflammation and apoptosis, respectively. It was also discovered that FA effectively prevented MPTP-induced neuronal loss-triggered declines in behavioral function and motor coordination [233]. Additionally, FA reduced proinflammatory cytokines and inflammatory mediators such as iNOS and COX-2. Further, the results confirmed that FA mitigates activation of microglial and astrocytes by a remarkable reduction in GFAP and Iba-1 hyperactivity [232]. In addition to their role in mitochondrial propagation and function, dynamin-related protein 1 (Drp1) and mitofusin 2 (Mfn2) have also been linked with excitotoxicity and are thought to play a vital role in programmed cell death [234]. In fact, enhanced expression of Drp1 has been linked to neuronal damage in animal models of PD [235]. Moreover, the study's author reported that FA administration attenuated 6-OHDA-induced morphological changes and DNA damage and blocked caspase activity. FA also reduced mitochondrial expression of Drp1 and increased expression of the PGC1*α* gene and protein, thereby regulating expression of its downstream target Mfn2 and restoring mitochondrial dynamics in 6-OHDA-lesioned PD animal models [171]. Hence, the combination of FA and muscle exercise effectively improved motor function and increased HSP-70 expression and TH-positive fibers in the corpus ST in rotenone-induced PD mice [172].

Sirtuin 2 (SIRT2) is a potential culprit in PD pathology and modulates the *α*-synuclein accumulation that is critical to several pathological processes in PD. Recent *in vitro* and *in vivo* studies using PD models have revealed that pharmacological inhibition of SIRT2 activity potentially ameliorates the *α*-synuclein-mediated toxicity reported previously [236]. Deacetylation of Nrf2 by SIRT2 leads to a reduction in both nuclear and total cellular levels of Nrf2 through its degradation [237]. Interestingly, FA treatment prevents MPTP-induced oxidative stress through activation of ERK1/2 signaling and inhibition of SIRT2, processes that are facilitated by independent mechanisms. Additionally, FA attenuated motor impairments in MPTP-injected *α*-synuclein knockout mice and wild-type mice, but not in Nrf2 knockout mice [173]. Therefore, these antioxidant and anti-inflammatory properties add to the value of FA as a therapy for PD.

### 6.5. Neuroprotective Mechanisms of Thymoquinone in PD

Thymoquinone (TQ) is a pharmacologically active compound found in black cumin seeds and plants from the Lamiaceae family [238]. Black cumin has been used in medicine since ancient times; more recently, interest in this compound has increased significantly [239]. Several investigations have demonstrated that black cumin seeds and their active constituent, TQ, may be suitable for clinical trials because most of the major effects of TQ have been shown to be beneficial. Intake of any black cumin seed has a recommended daily intake range of approximately 250–1000 mg [240]. Previous experimental studies have confirmed that TQ and its derivatives evince several pharmaceutical activities, including antioxidant, anti-inflammatory, antihypertensive, antiasthmatic, antidiabetic, and antitumor properties [241–243]. Interestingly, the effects of TQ were studied for the *α*-synuclein-induced synaptic toxicity in the cultures of rat hippocampal neurons and neurons differentiated from human induced pluripotent stem cells (IPSCs). In both types of cultures, TQ protected the neurons against *α*-synuclein-induced synaptic damage, increased the level of synaptophysin (a synaptic density marker), and prevented inhibition of synaptic vesicle recycling induced by the mutant *β*-synuclein (P123H). Moreover, using cells cultured on the multielectrode arrays, the authors demonstrated that TQ maintained normal bioelectrical activity in the neuronal network that was damaged by the actions of *α*-synuclein [244]. As previously described, autophagy is a natural cellular mechanism for eliminating unnecessary or damaged organelles and molecules and can be induced by oxidative or toxic stress. The disturbance of the autophagy mechanism can lead to the development of neurodegenerative diseases. TQ at a concentration of 0.0110 *μ*M prevented the MPP^+^-induced death of mesencephalic dopaminergic neurons *in vitro* by reducing the release of lactate dehydrogenase and maintaining MMP. The effect of TQ was accompanied by the activation of autophagy, which contributed to the reduction of the apoptotic neuron death [245].

The neuroprotective effect of TQ was demonstrated in a rotenone-induced PD model, in which TQ treatment prevented the death of primary dopaminergic neurons [246]. Furthermore, TQ significantly upregulated the expression of neuroprotective proteins, significantly downregulated the expression of proinflammatory cytokines, and inhibited activation of NF-*κ*B against LPS/IFN*γ*-activated BV-2 microglial cells [247]. Importantly, TQ manifested the ability to improve the course of PD in *in vivo* experiments. In mice with MPTP-induced PD accompanied by the development of oxidative stress and neuroinflammation in the brain, TQ restored the activities of anti-inflammatory enzymes, prevented GSH exhaustion, inhibited lipid peroxidation, and decreased levels of proinflammatory cytokines [174]. In a rotenone-induced PD model, TQ prevented the development of motor impairments and changes in the content of Parkin and Drp1 proteins and increased DA levels in the SN and ST areas of rat brain [175]. The neuroprotective action of TQ was observed in the animals after injection of 6-OHDA into the ST which led to the loss of neurons and behavioral functions [176].

### 6.6. Neuroprotective Mechanisms of Ellagic Acid in PD

Ellagic acid (EA) is a natural antioxidant phenol found in several vegetables and fruits, in particularly large quantities in persimmon, pomegranates, nuts, black raspberry, raspberry, peach, strawberry, and plumes [248]. There is a relatively high content of EA in raspberries (1500 mg/g dry weight), strawberries (630 mg/g dry weight), cranberries (120 mg/g dry weight), walnuts (590 mg/g dry weight), pecans (330 mg/g dry weight), and a number of other plant foods [249]. The daily intake of total polyphenols is the highest in Denmark at approximately 1786 mg/day, followed by Japan at 1500 mg/day. European countries and the Americas average about 900 mg/day and 800 mg/day, respectively. In Europe, Poland and France have the highest intake—after Denmark—at above 1000 mg/day; Italy averages approximately 650 mg/day, Greece about 584 mg/day, and Spain has the lowest intake at 300 mg/day [250]. In Asian countries, such as China and Korea, apples and vegetables seem to serve as the main sources of polyphenols, while green tea provides the highest intake for the Japanese population [251]. The appropriate dose of an EA supplement depends on several factors, including the age and health of the consumer. Currently, there are no standard dosing recommendations for EA.

Moreover, EA has been shown to have potentially antioxidant, anti-inflammatory, antiviral, antisteatotic, antibacterial, antihepatotoxic, anticholestatic, anticancer, antidiabetic, immunomodulatory, and antiproliferative properties, representing an extensive range of beneficial effects that may be applied to improve human health [248, 252–254]. The bioavailability of EA is low compared with that of other phenols; however, punicalagin, which is a source of EA, was detected in human plasma after consumption of pomegranate juice [255, 256]. Evidence indicates that pomegranate juice containing 318 mg of punicalagin and 25 mg of EA consumption led to a plasma concentration of 31–33 ng/mL (maximum concentration) EA 1 h after absorption [255, 256]. After oral administration to rats, both punicalagin and EA were detected in the plasma after consumption of pomegranate leaf extract [257–259].

Several preclinical trials have suggested that EA effectively attenuates renal oxidative damage in LPS/D-galactosamine-induced PD rats by reducing the levels of lipid peroxidation and boosting the antioxidative defense system via increased levels of enzymic and nonenzymic antioxidant activities [260]. Moreover, treatment with EA enhanced the GSH content and mRNA expression of renal and hepatic SOD and GPx activity. In addition, EA treatment also prevented elevated renal and hepatic levels of MDA and NO production [261]. Of note, EA administration prevented 6-OHDA-induced increased levels of MDA and decreased GPx and SOD activities in both ST and hippocampus tissues. EA treatment also improved motor function and electrophysiological performance in medial forebrain bundle-lesioned rats by raising cerebral antioxidant content [177]. Other studies discovered that combined treatment with three compounds such as EA, *α*-lipoic acid, and myrtenal attenuated decreases in DA levels, substantially decreased lipid peroxidation levels, and restored CAT activity in 6-OHDA-induced PD rats [262].

Interestingly, a study involving the kinetics of enzyme inhibition demonstrated that EA combined with curcumin inhibited MAO-B activity via both competitive and noncompetitive inhibition [263]. Importantly, previous research has shown that dimethyl fumarate, a pharmacological activator of Nrf2 that is presently used in multiple sclerosis, may also exert beneficial effects in PD [264]. EA was also utilized to confer neuroprotection against rotenone-induced neurotoxicity through activation of Nrf2 signaling, which was involved in EA-mediated DA neuroprotection [265]. Furthermore, similar research also revealed that EA reduced striatal MDA levels, ROS, and DNA fragmentation and improved Nrf2, HO-1, and behavioral functions. Meanwhile, EA prevented the loss of TH-positive neurons within the SN in 6-OHDA-induced rat models of PD [178]. These results suggest EA is a potential Nrf2 activator, and that by restoring antioxidant mechanisms, EA may serve as a promising therapeutic candidate for PD in the future.

### 6.7. Neuroprotective Mechanisms of Caffeic Acid in PD

Caffeic acid (CA) is a phenolic acid extracted from numerous plant species and is present in beverages such as coffee, wine, and tea. CA carries medicinal properties such as those found in propolis, which has been studied extensively for its strong antioxidant potential [266–269]. Emerging epidemiological evidence suggests that greater coffee consumption may reduce the risk of NDDs. The daily intake of CA has been estimated to be about 500–1000 mg in humans consuming fruits, vegetables, beer, and coffee [226]. Drinking a single cup of coffee provides as much as 70-350 mg of chlorogenic acid or CA [270]. Recent evidence revealed the highest category of coffee consumption (Netherlands, 6 cups/day; Sweden, 2-5 cups/day; Spain, 1-2 cups/day; Japan, 3-5 cups/day; Italy, 1-3 cups/day; Finland, 1-4 cups/day; and China, 1-3 cups/day) [33, 271].

CA has been reported to have several pharmacological activities, including anticancer, anti-inflammatory, and neuroprotective properties [180, 272]. It has also been reported that CA may react with peroxyl radicals involved in lipid peroxidation to efficiently mitigate several disease conditions of PD [180, 273]. CA is believed to remove excess ROS/RNS generation, and CA is known to stimulate antioxidative enzyme activities including SOD, GPx, GSH, and CAT [273]. In PQ-induced fruit fly, using a *Drosophila melanogaster* model, a molecular docking study demonstrated the strong interaction of CA with *Drosophila melanogaster* transcriptional activation of Nrf2. In observations of the binding of CA to the Keap1 domain of Nrf2, results show that the protective effect of CA reduction of lipid peroxidation level and ROS in *Drosophila melanogaster* was possible through its coordination, which delays Nrf2-Keap1 binding and leads to an enhanced antioxidant defense system [179]. In the 6-OHDA-induced SH-SY5Y cellular model, CA-phenethyl ester significantly improved cell viability, diminished apoptotic cell death, and prevented changes in damaged nuclear morphology. Furthermore, treatment with CA-phenethyl ester could maintain mitochondrial function, inhibit ROS production, upregulate Bcl-2 and Akt levels, and downregulate Bax expression [274]. Similarly, in MPP^+^-induced neurotoxicity *in vitro* PD models, CA-phenethyl ester directly inhibited release of Cyto-C and apoptosis-inducing factor (AIF) from mitochondria [275].

Furthermore, one highlight resulting from CA administration was a significant increase in the efficiency of striatal DA content and, consequently, a decrease in microglia expressions and inflammatory mediators. In addition, histopathological assessment of SN neurons demonstrated high immunostaining for TH-positive cells and improved motor performance in rotenone-treated mice [276]. In seven-month-old A53T *α*-synuclein transgenic mice, CA alleviated cell damage and reduced A53T *α*-synuclein expression by activating the JNK/Bcl-2-mediated autophagy signaling pathway. It was also observed that the CA's protective efficacy on A53T *α*-synuclein degradation was prevented by the JNK inhibitor SP600125 and the autophagy inhibitor bafilomycin A1. In A53T Tg mice, CA attenuated loss of dopaminergic neurons, improved behavioral function, enhanced autophagy activation, and reduced *α*-synuclein accumulation in the SN [180]. In MPTP-induced PD mice, CA lowered the production of proinflammatory cytokines, diminished the expression of inflammatory mediators and GFAP, and decreased the production of NO and PGE_2_. In addition, CA restored the expression of BDNF and GDNF and maintained TH-positive cells and DA synthesis [181]. Previous research discovered that metallothionein- (MT-) 1 and MT-2 were upregulated, particularly in ST astrocytes, by activation of Nrf2 signaling in response to oxidative stress and acted to protect SN neurons [277]. Treatment with CA protected both SN and intestinal enteric neurons and upregulated MT-1 and MT-2 antioxidative molecules in the ST astrocytes of rotenone-induced C57BL/6J mice [278]. Interestingly, the inhibitory effect of CA against escitalopram-induced *α*-synuclein accumulation and other neurotoxins may prove to be promising novel therapeutic drugs for PD [279].

### 6.8. Neuroprotective Mechanisms of Epigallocatechin-3-Gallate in PD

Epigallocatechin-3-gallate (EGCG), a polyphenol isolated from green tea, is known for its myriad physiological beneficial effects against inflammatory disorders, cancer, and NDDs in humans [280–282]. Several pharmacological activities including antioxidant, anti-inflammatory, metal-chelating, radical-scavenging, antiapoptotic, and anticarcinogenic properties by modulation of several transcription factors, proteins, and other important growth factors are attributed to EGCG [282, 283]. EGCG's broad potential for improving healthy aging derives from its promotion of morphologic and functional alterations that occur naturally in an aging brain; these alterations increase memory and learning ability, suppress cognitive dysfunction, and reduce oxidative damage in the brain [284–286]. The typical daily intake of EGCG resulting from the consumption of green tea infusions ranges from 90 to 300 mg/day, while exposure in high-level consumers is estimated to be as much as 866 mg EGCG/day in the adult population in Europe. Food supplements containing green tea catechins provide a daily dose of EGCG in the range of 5–1000 mg/day for the adult population. Based on available data on the potential adverse effects of green tea catechins on the liver, the evidence from interventional clinical trials of doses equal to or above 800 mg/day in the form of a food supplement has shown increased bioavailability of EGCG in treated subjects compared to control [287–290]. A few studies have previously demonstrated that EGCG can cross the BBB easily, which is crucial for the development of therapeutic agents for PD [291, 292].

Several studies of human subjects have described an inverse dose-response relationship between green tea consumption and cognitive impairments in PD disorders [293, 294]. Recent advancements in case-controlled joint studies revealed that daily consumption of two or more cups of tea reduced cognitive dysfunction and decreased the prevalence of PD [295, 296]. A Finnish longitudinal study conducted over 13 years with 30 000 adults aged 25–74 years old showed that daily consumption of three or more cups of tea reduced the risk of PD [297]. Similarly, a large-scale-cohort, 20-year follow-up study involving nearly 50 000 male and 80 000 female volunteers demonstrated that EGCG intake was associated with a 40% lower risk of PD [298]. Moreover, several cohort- and case-controlled studies across Asian, European, and North American countries have reported a link between tea consumption and lower risk of PD [299]. In Asian populations, particularly among the Chinese, 28% of populations experienced a decreased risk of developing PD with daily consumption of three cups of tea for ten years [299].

Currently, several findings have demonstrated the antioxidative capacity of EGCG under experimental conditions in both cellular and animal models [300, 301]. In rotenone-induced rat PD models, EGCG treatment reduced lipid peroxidation production and NO levels, increased succinate dehydrogenase (SDH) activity, improved mitochondrial function, and raised ATPase and ST catecholamine levels. In addition, EGCG treatment decreased the level of neuroinflammatory cytokines and apoptotic markers and improved motor performance [302]. Administration of EGCG in MPTP-induced mice prevented neurotoxin-induced reduction in ST antioxidant enzymes SOD and CAT and increased the activities of both enzymes in the total brain [303]. Furthermore, EGCG treatment wholly prevented STAT3 activity and stimulated neuronal cell proliferation induced by 6-OHDA in SH-SY5Y cells [304]. Most recent findings have confirmed that the neurorescue effect of EGCG regulated the iron-export protein ferroportin in the SN, reduced oxidative stress, and attenuated functional and neurochemical shortages against MPTP-induced PD mice [182]. In the MPTP-injected mouse model, EGCG restored movement behavior and protected TH-positive cells in the SN region. Flow cytometric analysis showed that the ratio of CD3_+_CD4_+_ to CD3_+_CD8_+_ T-cell lymphocytes in the peripheral blood increased with EGCG treatment and reduced expression of inflammatory factors such as TNF-*α* and IL-6 in the serum [183]. Furthermore, EGCG was shown to display antiapoptotic effects in PQ-induced PC12 cell models: EGCG maintained MMP and inhibited the upregulation of caspase-3 activity and the downregulation of the proapoptotic SMAC protein in cytosol expression [305]. It was also observed that EGCG treatment reduced TNF-*α* and NO inflammatory mediators and attenuated loss of midbrain DA levels triggered by LPS-induced neurotoxicity [306]. Furthermore, *in vitro* and *in vivo* studies demonstrated that cotreatment with EGCG lowered glutamate-induced oxidative cytotoxicity in HT22 cells through inhibition of NF-*κ*B activation. In addition, EGCG treatment moderated the effect of decreased accumulation of 3-O-methyldopa in the plasma and ST of rats that were administered carbidopa+L-Dopa; EGCG also exerted a strong therapeutic effect against kainic acid-induced oxidative neuronal death in the hippocampus of PD rats [307].

The process of *α*-synuclein and other protein deposition has been strongly linked with numerous NDDs, including PD. Recently, several studies have attracted attention by showing that *α*-synuclein accumulation formation might be mediated by small molecules, such as the polyphenol EGCG, which may offer a potential therapeutic option for management of *α*-synucleinopathies [308, 309]. Other research found that EGCG potentially interacted with *α*-synuclein amino acid sites found on peptide membranes. It was implicated that EGCG binds to *α*-synuclein via unstable hydrophobic interactions; these findings support the assertion that EGCG could be a potent remodeling agent of *α*-synuclein accumulation and a potential disease-modifying agent for the treatment of PD [184].

### 6.9. Neuroprotective Mechanisms of *α*- and *β*-Asarone in PD

Alpha- (*α*-) asarone and beta- (*β*-) asarone compose an important antioxidant aromatic chemical constituent that is extracted from the rhizomes of *Acorus calamus*. Consequently, both *α*- and *β*-asarone have been reported to have one or more similar pharmacological properties that may offer beneficial effects in the therapeutic management of several diseases [310–313]. Importantly, the delivery of *α*- and *β*-asarone in the brain is extensive, demonstrating its ability to cross the BBB, a desirable characteristic of compounds used for the treatment of numerous NDDs [310]. A recent Swiss ADME predictor study revealed that absorption, distribution, metabolism, and excretion results showed that *α*- and *β*-asarone possess good oral bioavailability; the study also showed a good binding affinity towards dopaminergic receptors. Further, *α*- and *β*-asarone were found to interact with different amino acid residues of disease-modifying D2 and D3 receptors through hydrogen bonding [314].

In one PD model, *α*-asarone treatment reduced neural inflammation and suppressed IL-*β*, IL-6, and TNF-*α* production in LPS-stimulated BV-2 cells. In addition, *α*-asarone treatment effectively inhibited the LPS-stimulated activation via regulation of NF-*κ*B by blocking degradation of inhibitor NF-*κ*B signaling in BV-2 microglial cells. *In vivo* studies also demonstrated that prophylactic administration with *α*-asarone inhibited microglial activation and attenuated PD-like behavioral deficits in MPTP-injected PD mice [185]. In the 6-OHDA-induced PD model, *β*-asarone improved the behavioral function of rats in the initiation time, open field, stepping time, and rotarod tests. Research has also found that *β*-asarone increases the levels of HVA, DOPAC, and 5-HIAA in the ST region. In addition, administration with *β*-asarone elevated the level of TH-positive neurons and inhibited the expression of LC3-II in SN4741 cells. Moreover, *in vivo* experimental results showed that *β*-asarone affected the expression of Bcl-2, Beclin-1, JNK, and p-JNK in 6-OHDA-injected PD rats. The neuroprotective effect of *β*-asarone occurs primarily by downregulating JNK and p-JNK expressions and then indirectly increasing Bcl-2 expression. Additionally, *β*-asarone may inhibit the function of Beclin-1, thereby inhibiting autophagy activation [186]. Activated autophagy is an important process that may play a defensive role through clearance of toxic aggregated *α*-synuclein in neurons [315]. On the other hand, dysfunction of the autophagy-lysosomal pathway has been associated with the development of PD [316]. Recent studies also proposed that endoplasmic reticulum (ER) stress may induce autophagy [317]. In 6-OHDA-induced PD rat models, *β*-asarone administration may decrease the levels of Beclin-1, CHOP, GRP78, and p-PERK while significantly increasing the level of Bcl-2. *β*-Asarone may increase Bcl-2 by inhibiting the p-ERK pathway, and Bcl-2 may inhibit the expression of Beclin-1. The results of that study suggested that *β*-asarone may regulate autophagy and ER stress via the PERK/CHOP/Bcl-2/Beclin-1 pathway [187]. Very recent findings have shown that *β*-asarone can effectively inhibit neuronal apoptosis through the CaMKII/CREB/Bcl-2 signaling pathway and regulate Bcl-2 family proteins [318]. Moreover, *β*-asarone significantly lowered the expression levels of MALAT1 and *α*-synuclein in the midbrain of MPTP-injected PD mice. In addition, immunoprecipitation and RNA pull-down assays confirmed that MALAT1 was associated with *α*-synuclein, leading to the increased stability of *α*-synuclein and its expression in SH-SY5Y cells. *β*-Asarone treatment could increase the viability of cells exposed to MPP^+^[319]. Similarly, another study demonstrated that *β*-asarone exerted antioxidative effects on H_2_O_2_-stimulated PC12 cells by reducing oxidative stress via activation of the protective Nrf2/HO-1 pathway [320].

Based on the pharmacological effect of *β*-asarone, previous scientific studies demonstrated that combined treatment with *β*-asarone and L-dopa carried potentially therapeutic value. Interestingly, combined treatment with *β*-asarone and L-dopa reduced the level of creatinine and increased the level of HVA, DA, DOPAC, and 5-HT, while *β*-asarone also enhanced TH and DAT protein expression in madopar-induced PD rats [321]. Similar findings also show that LC3B and Beclin-1 expression decreased, while p62 expression increased after coadministration with *β*-asarone and L-dopa. In addition, the group that received coadministered *β*-asarone and L-dopa exhibited a significant decrease in autophagosome activity when compared with the 6-OHDA-injected PD control group [322]. However, further experimental validation using *in vitro* and *in vivo* studies is needed before clinical trials may commence.

### 6.10. Neuroprotective Mechanisms of Theaflavin in PD

Theaflavin (TF) is representative of a group of polyphenols that are found in black tea, comprising theaflavin-3-gallate, theaflavin-3′-gallate, and theaflavin-3,3′-digallate, which contribute to the quality and color of black tea [323]. TF is known for its several therapeutic effects owing to its antioxidant properties: removal of excess free-radical formation and metal chelation ability [324–327]. In the past few years, several scientific reports have shown that TF has potential neuroprotective effects against NDDs. TF has been found equal in efficiency to EGCG at inhibiting *β*-amyloid- and *α*-synuclein-induced neurotoxicity due to its potential antioxidant properties [328]. Recently, a randomized, double-blind, placebo-controlled study showed that the beneficial TF doses of 50 mg/day and 100 mg/day provide health benefits. These TF doses also appeared to be more effective than similar doses of tea catechin [329]. Recently, a clinical survey from the European Union demonstrated that the daily intake of TF ranged from 181 mg/day (Czech Republic) to 793 mg/day (Ireland). The highest intakes of TF were observed in Ireland (191–505 mg/day) and the lowest intakes in Spain (9–24 mg/day) [330]. Little data exists on the pharmacokinetic profile of TF in humans: after the consumption of 700 mg of TF once a day, corresponding to about 30 cups of black tea, achieved maximum concentration observed in blood plasma and betterment of oral bioavailability [331].

Cell lines were used to investigate the mechanism of action of TF, 6-OHDA-induced SH-SY5Y, and the findings revealed attenuated loss of cell viability, reduced nuclear morphology, decreased apoptosis, increased MMPs, and diminished intracellular NO levels. These results suggested that TF had a protective effect against 6-OHDA-induced apoptosis through inhibition of NO and ROS production [332]. In the PC12 cell line treated with H_2_O_2_, oxidative stress was eliminated by administration of TF, which decreased Bax and caspase-3 protein expression and increased Bcl-2 expression. This finding indicates that TF possesses antiapoptotic properties, providing both cytoprotection and neuroprotection [333]. Another study demonstrated that TF acted as a potent inhibitor of *β*-amyloid and *α*-synuclein fibrillogenesis and stimulated the *β*-amyloid and *α*-synuclein assembly into a nontoxic form. These results suggest that TF could be used to remove toxic amyloid deposits [328].

In the PD mouse model, TF increased the expression of DAT and VMAT-2 in addition to downregulating the effects of oxidative stress in MPTP-induced neurotoxicity in mice. TF has also been shown to ameliorate dopaminergic neuronal loss and behavioral deficits [188]. In mice treated with MPTP/p, TF administration increased the expression of nigral TH and DAT and lowered caspase-3, caspase-8, and caspase-9; these results were accompanied by increases in regulated behavioral function [189]. Moreover, dysfunction of the cholinergic system was also observed to trigger the production of proinflammatory cytokines and activation of microglia: the levels of IL-4 and IL-10 anti-inflammatory markers were raised in MPTP-injected mice as a compensatory mechanism against neuronal inflammation [334]. In a recent *in vivo* study, treatment with TF ameliorated the chronic MPTP-induced neurotoxicity in the SN and ST of mice, as evidenced by significantly decreased neuroinflammation and apoptosis. In addition, treatment with TF attenuated the MPTP-injected behavioral impairments such as catalepsy and akinesia and significantly reduced the excess formation of IL-4 and IL-10 anti-inflammatory cytokines [190].

## 7. Neuroprotective Roles of Antioxidant Phytochemicals and Their Analogs

As the present study has explained, some naturally derived phytochemical constituents are potential neuroprotective agents that may have applications for treating PD. The chrysin derivatives 6,8-bis(*o*-tolylselanyl)-chrysin, 6,8-bis(*p*-anisoylselanyl)-chrysin, and 6,8-bis(*p*-fluorophenylselanyl)-chrysin were semisynthesized and studied for their antioxidant and neuroprotective activity [335]. The author of that study suggested that the structure-activity relationship, with all three compounds containing a fluorine atom in the *para* position to selenium, showed the greatest antioxidant activity through its ability to inhibit lipid peroxidation and ROS generation in mouse cortex and hippocampus. Scipioni et al. [336] demonstrated that synthesized novel vanillin derivatives of 4,4′-(((3-hydroxypropyl)azanediyl)bis(methylene))bis(2-methoxyphenol) and 4,4′,4^″^,4^‴^-((1,4-phenylenebis(azanetriyl))tetrakis(methylene))tetrakis(2-methoxyphenol) having a tertiary amino group, accompanied by the number of vanillin moieties, have confirmed ability to protect from oxidative damage and potential antioxidant activity in H_2_O_2_-induced neuroblastoma SH-SY5Y cells. Another study reported that semisynthesized AA derivatives including AS-2, AS-2-9-006, and AS-9-006 exhibited the greatest activity in the active avoidance, passive avoidance, and the Morris water maze tests and cognitive-enhancing activity [337]. 1-Feruloyl glycerol (FA-G1) and 1-feruloyl diglycerol (FA-DG1), two water-soluble derivatives of FA, exhibited neuroprotective effects against *β*-amyloid-induced neurodegeneration in both *in vitro* and *in vivo* experiments. This neuroprotection was evidenced by inhibition of NO production and reduction in iNOS expression in a dose-dependent manner mediated by suppression of NF-*κ*B nuclear translocation in primary astrocytes, by beneficial effects against abnormal activation of astrocytes, and by a reduction in neurodegeneration [338].

A very recent study demonstrated that lipophilic butyl ferulate, a derivative of FA, binds to amide NH in Gln15 and Lys16 via a hydrogen bond. This binding significantly attenuated intracellular ROS formation and could potentially upregulate antioxidant enzyme activity by modulating the Keap1-Nrf2-ARE signaling pathway [339]. Another study reported the neuroprotective effect of two CA derivatives known as caffeic acid phenethyl ester and danshensu ((R)-4 2-hydroxy-3-(3,4-dihydroxyphenyl)propionic acid), which contain numbers of hydroxyl groups in an aromatic ring (A) that are conjugated with double bond. These study results demonstrated that these two compounds significantly boost the endogenous antioxidant defense system and modulation of the PKA/CREB signaling pathway. Furthermore, these compounds significantly improved behavioral performance in both the step-down avoidance test and Morris water maze test [340]. A similar study also revealed that CA-phenethyl ester 4-O-glucoside, which is synthesized from CA, suppressed H_2_O_2_-induced oxidative stress by inhibiting ROS generation, protein carbonylation, and MDA content, in addition to significantly enhancing GSH and SOD activities in both SH-SY5Y and PC12 cells. Mechanistically, it prevented impairments in learning and cognition *in vivo* by reducing neuronal cell death and protecting against hippocampus and cortex dysfunction [341]. The most promising polyphenolic TF derivatives, namely, TF-3-gallate, TF-3′-gallate, and TF-3,3′-gallate, exhibited significant rescue from the metabolic inhibition induced by *α*-synuclein aggregates in PC12 cells [328].

## 8. Neuroprotective Role of Phytochemical Nanoformulation

Several nanodelivery systems loaded with naturally derived antioxidant phytochemicals have been demonstrated to be effective in modulating oxidative stress, *α*-synuclein aggregation, chronic inflammation, and various signaling pathways that mediate most aging-associated NDDs, particularly in PD. For instance, in a study by Giacomeli et al. [342], chrysin-loaded lipid-core nanocapsules showed higher antioxidant ability and reduced neurotoxicity through reduction of oxidative stress and neuroinflammation and through modulation of neurochemical and behavioral changes in an aged animal, compared with free chrysin. A very recent study observed that chrysin-loaded poly (lactic-co-glycolic acid) nanoparticles less than 150 nm in size in pentylenetetrazol-induced epilepsy mice showed chrysin nanoparticle treatment counteracted oxidative stress, reduced neuronal apoptosis, and upregulated Nrf2, HO-1, and NAD(P)H quinone oxidoreductase [343]. Trans-FA-loaded solid lipid nanoparticles could enable the uptake of FA by cells due to of their lipophilic nature, thereby increasing FA bioavailability and concentration-dependent reduction of lipid peroxidation and enhancement of antioxidant enzyme activities in rat brain [344]. Anti-inflammatory effects of FA-loaded nanoparticles such as modified glycol chitosan nanoparticles have also been reported previously [345]. Another study demonstrated that rats that received intravenous injections of an FA-loaded nanostructured lipid carrier exhibited significantly attenuated neurobehavioral deficits, oxidative stress, and cellular damage; the study showed that activating the PI3K pathway may be of beneficial effect in cerebral stroke [346].

There is evidence that nanoencapsulated TQ carry greater antioxidant and neuroprotective properties. In a rat model, TQ-loaded mesoporous silica nanoparticles 90 nm in size and spherical in shape were able to cross the BBB. Results showed that the encapsulated TQ-loaded mesoporous silica nanoparticles enhanced drug target delivery to all brain areas (ST, cortex, thalamus, midbrain, and hypothalamus) and significantly reduced oxidative stress biomarkers [347]. Numerous studies have reported that different nanoparticle-loaded phytochemicals (e.g., vitamin E, resveratrol, curcumin, and hyaluronic acid) with an average particle size of 100 nm resulted in higher ROS scavenging efficiency and lower lipid peroxidation in patients with PD [348–350]. Similarly, TQ-loaded PLGA-chitosan nanoparticles (particle size from 183 nm) delivered via the nose-to-brain route in rodents improved their pharmacokinetic profile in the brain and enhanced grip strength and locomotor activity. In addition, these effects were supported by a significant reduction in levels of lipid peroxidation and increase in antioxidant enzyme activity in the brain of middle cerebral artery-occluded rats [351]. The anti-inflammatory and neuroprotective potential of stress-induced TQ-loaded solid lipid nanoparticles has been demonstrated by significantly enhanced antidepressive-like behavioral function, hippocampus BDNF levels, and reduced levels of hippocampal IL-6 and TNF-*α* expression compared with free TQ [352]. TQ-loaded solid lipid nanoparticle treatment also attenuated the overexpression of GFAP, proinflammatory cytokines, and p-p65 NF-*κ*B nuclear translocation; improved the number of TH-positive neurons; and ameliorated motor deficits in neurotoxin-intoxicated animals when compared with the free TQ-treated group [353].

Recently, El-Missiry et al. [354] studied the neuroprotective and antiepileptic efficacy of EA-loaded calcium-alginate nanoparticles (sized approximately 150 nm) in pentylenetetrazol-induced seizures in male mice. These EA-loaded calcium-alginate nanoparticles were also able to ameliorate oxidative stress, as evidenced by enhanced antioxidant efficiency and decreased 4HNE levels in the brain. Furthermore, the nanoformulation outperformed free EA in several activities: amelioration of apoptosis, inhibition of Cyto-C release, activation of caspases, regulation of P53, Bax, and Bcl-2 protein expression, and protection against DNA damage. Moreover, the EA and chitosan-coated combination mitigated rotenone-induced ROS overproduction and reduced cytotoxicity [355]. The antioxidant and antiaging properties of CA-loaded nanotransfersomes were found to enhance cell viability, reduce intracellular ROS generation, attenuate lipid peroxidation, and modulate MMP expression [350]. In addition, anticonvulsive and neuroprotective effects of EGCG-loaded PEGylated-PLGA nanoparticles (particle size from 169 nm) safe for brain cells significantly reduced neuroinflammatory marker expression and were capable of increasing drug integrity and bioavailability [356]. The *α*-asarone-loaded lactoferrin-modified mPEG-PLA nanoparticles delivered intranasally to the brain showed increased nasal permeability, brain targeting, and brain systemic exposure and reduced toxicity without affecting bioavailability [357].

## 9. Conclusion

In recent years, research into naturally derived phytochemicals targeting several pathogenic conditions of age-associated NDDs has increased, and clinically, no serious adverse reactions of currently available phytochemicals have been documented. Moreover, PD has been therapeutically cured by natural products, in particular by naturally food-derived phytochemicals with antioxidant potential that may present a reliable source of medicine. The clinical evidence of the health benefits of phytochemicals is not yet fully accepted. However, naturally derived lipophilic phytochemicals can enter the brain and cross the BBB easily, offering increased bioavailability, faster metabolism, and higher affinity to receptors. The regular administration of these naturally derived phytochemicals is an imperative approach to enhance the reversal of neuron function decline and disease resistance competency. Much of the *in vivo* experimental evidence presented in the current study of the potential neuroprotective effects of antioxidant phytochemicals was supported by the results of histopathological and immunohistochemical investigation, which indicated the protection of dopaminergic neurons and attenuation of the loss of TH-positive cells. Furthermore, these changes were accompanied by improvement of several neurotoxin-induced motor balance and cognitive deficits of study animals, as evaluated by rotational behavior, open field test, beam walking, and horizontal and vertical grid tests, as well as by passive avoidance, Barnes maze, and other nonmotor behavioral patterns.  Table 1 provides a summary of well-recognized and reported antioxidant phytochemicals and their molecular mechanisms of action in PD.

**Figure 3:**
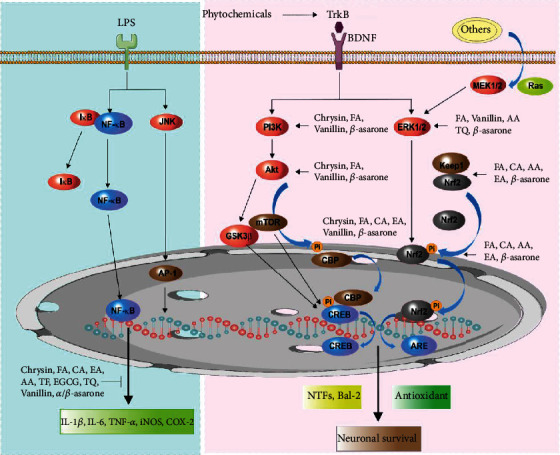
Intracellular targets of neuroprotective antioxidant phytochemicals by activation of Keap1/Nrf2/ARE signaling pathways to increase the expression of antioxidant enzymes. The modulation of these pathways by natural antioxidant phytochemicals such as chrysin, vanillin, asiatic acid (AA), ferulic acid (FA), thymoquinone (TQ), ellagic acid (EA), caffeic acid (CA), epigallocatechin-3-gallate (EGCG), *α*- and *β*-asarone, and theaflavin (TF).

Collectively, the antiparkinsonian effects of antioxidant phytochemicals have been demonstrated in the reports cited in the present study. It is known that oxidative stress and neuroinflammation are important factors responsible for the progression of PD. Hence, our study provides evidence that the anti-inflammatory activities of antioxidant phytochemicals offer a safe approach to protect against the neuronal damage by reducing oxidative stress, inhibiting lipid peroxidation activity, enhancing the content of GSH, modulating the secretion of proinflammatory cytokines, regulating inflammatory mediators such as COX-2, iNOS, and NO, and regulating anti-inflammatory molecules and pathways (Figure 3). Abundant evidence suggests that oxidative stress and *α*-synuclein accumulation trigger activation of microglia and astrocytes; this activation is associated with the complex neuroinflammatory pathways leading to neurodegeneration in PD. A number of antioxidant phytochemicals have been shown to clear *α*-synuclein accumulation and to inhibit microglial activation accompanied by suppressed IBA-1 and GFAP expression. Simultaneously, several antioxidant phytochemicals discussed enhanced the expression of GDNF and BDNF levels involved in the survival of DA neurons. Recently, several experimental findings demonstrated that the JNK signaling pathway involved in apoptotic actions belonging to the superfamily of MAPKs responds to induced ROS and plays a significant role in triggering apoptosis. Several antioxidant phytochemicals are being directed towards some molecular pathways underlying the neuroprotective properties; thus, phytochemicals have been observed to restore the reduced level of antiapoptotic Bcl-2, lowering the expression of proapoptotic Bax and inhibiting caspase activity. Furthermore, phytochemicals have been confirmed to reduce JNK activation and transcription factor c-Jun, resulting in the reduction of dopaminergic neuron apoptosis.

Together, oxidative stress, neuroinflammation, and Nrf2/ARE deregulation are common major situations in the pathogenesis of NDDs, particularly in PD, resulting in impaired motor function and neuronal cell death. Moreover, some important modulators of Nrf2/ARE pathways and autophagy are altered in PD. Again, neuroprotection by antioxidant phytochemicals is associated with the activation of Nrf2/ARE pathways and autophagy signaling, which appear to be the most well-studied mechanisms for PD treatments. Moreover, inhibition of NF-*κ*B activation should be investigated further as a useful therapeutic approach to the treatment of PD. Accumulated data strongly suggest that antioxidant phytochemicals' potential for activating Nrf2/ARE pathways and autophagy signaling, shown to enhance the expression of Nrf2/ARE and autophagy-related genes, proves protective in several experimental models of PD (Figure 3).

In summary, naturally derived phytochemicals and their derivatives play a potential neuroprotective role in their multidimensional ability to regulate and modulate chronic inflammation, oxidative stress, and downstream signaling, the hallmarks of PD. In addition, to prevent the occurrence of NDDs and their threat to the population, it is essential to explore novel interventional procedures in the clinic for direct employment of dietary phytochemicals as supplements in everyday use. Moreover, the evident lack of toxicity and easy availability from natural resources highlight their advantages in adopting them in the diet. Future research should be aimed at increased clinical acceptance of claims from *in vitro* and *in vivo* preclinical studies and further clinical trial studies of several more potential compounds and the combinations thereof, to observe and prevent any undesirable side effects. The success of phytochemicals in clinical research will thereby be decisive in the evaluation of their pharmacological relevance in humans, and nutritional intervention programs will thereby decrease oxidative neuroinflammatory damage and reduce/slow down the progression of PD.

## Figures and Tables

**Table 1:** Promising studies of antioxidant phytochemicals for the management of PD.

Phytochemicals	Studied materials	Dose	Neurotoxins	Physiological effects	References
Chrysin	Male C57BL/6J mice	10 mg/kg	6-OHDA	↑Behavioral functions ↑TH-positive cells in the SN and ST ↑DA, DOPAC, and HVA levels	[151]
	Male C57BL/6 mice	50 and 100 mg/kg	MPTP	↑DA and its metabolites ↑AKT/GSK3*β*/MEF2D pathway ↓MAO-B activity	[152]
	Male C57BL/6J mice	50, 100, and 200 mg/kg	MPTP	↑BDNF and GDNF protein expression ↓IL-10, IL-6, TNF-*α*, and NF-*κ*B protein expression	[153]
Vanillin	Male Wistar albino rats	5, 10, and 20 mg/kg	LPS	↓iNOS, COX-2, IL-1*β*, and IL-6 protein expression ↓ERK1/2, p38, and NF-*κ*B signaling ↓Microglia activation	[167]
	Male Wistar albino rats	5, 10, and 20 mg/kg	Rotenone	↑Striatal DA and its metabolite levels ↑Behavioral function ↓Cyto-C, Bax, and caspase protein expression ↑Bcl-2 protein expressions	[168]
Asiatic acid	Male C57BL/6 mice	20, 40, and 80 mg/kg	MPTP	↑Striatal DA levels ↑Striatal TH, TLR4, BDNF, and GFAP protein expression ↓*α*-Synuclein and lowered AIF protein expression	[169]
	Male Wistar albino rats	100 mg/kg (*in vivo*) and 0.1–10 nM (*in vitro*)	MPTP/p and MPP^+^	↑Motor functions ↑PI3K, Akt, GSK-3*β*, and mTOR phosphorylation ↑TrKB protein expression ↓NLRP3 inflammasome expression in microglia cells	[170]
Ferulic acid	Male Wistar albino rats	100 mg/kg	6-OHDA	↓Mitochondrial Drp1 expression ↑PGC1*α* gene and protein expression ↑Mfn2 and mitochondrial dynamics	[171]
	Male C57BL/6 mice	100 mg/kg	Rotenone	↑Motor function ↑HSP-70 protein expression ↑TH-positive fibers in corpus striatum	[172]
	Male C57BL/6 mice	20 mg/kg and muscle exercise	MPTP	↑Motor behavior ↑CAT, SOD, GPx, and GSH activity ↓TBARS activity ↑Activation of the Nrf2 signaling	[173]
Thymoquinone	Male C57BL/6 mice	40 mg/kg	MPTP	↑CAT, SOD, GPx, and GSH activity ↓TBARS activity ↓iNOS, COX-2, IL-1*β*, and IL-6 protein expression	[174]
	Male Wistar albino rats	7.5 and 15 mg/kg	Rotenone	↑Behavioral functions ↑Parkin, Drp1, TH-positive cells in the SN and ST ↑ DA, DOPAC, and HVA levels	[175]
	Male Wistar albino rats	5 and 10 mg/kg	6-OHDA	↑Behavioral functions ↑DA level in the SN ↓MDA level	[176]
Ellagic acid	Male Wistar albino rats	50 mg/kg	6-OHDA	↑Motor function and electrophysiological performance ↑CAT, SOD, GPx, and GSH cerebral activity	[177]
	Male Wistar albino rats	50 mg/kg	6-OHDA	↑CAT, SOD, GPx, and GSH cerebral activity ↓TBARS activity ↓MAO-B activity ↑ER*β*/Nrf2/HO-1 signaling cascade	[178]
Caffeic acid	Drosophila melanogaster	0.5, 1, and 2 mg/g	Paraquat	↑CAT, SOD, GPx, and GSH cerebral activity ↓TBARS activity ↑Nrf2-Keap1 signaling	[179]
	A53T transgenic mice	5 mg/kg	A53T Tg mice	↓A53T *α*-synuclein ↑Bcl-2-mediated autophagy pathway ↑Behavioral functions	[180]
	Male C57BL/6 mice	0.5, 1, and 2 g/kg	MPTP	↑DA synthesis ↑TH-positive cells ↑BDNF and GDNF protein expression, maintained loss ↓IL-1*β*, IL-6, TNF-*α*, iNOS, and COX-2 expression ↓GFAP protein expression	[181]
Epigallocatechin-3-gallate	Male C57BL/6 mice	50 mg/kg	MPTP	↑Iron-export protein ferroportin in SN ↑CAT, SOD, GPx, and GSH cerebral activity ↓TBARS activity ↑DA synthesis	[182]
	Male C57BL/6J mice	25 mg/kg	MPTP	↑Movement behavior ↑TH-positive cells in the SN region ↑CD3^+^CD4^+^ to CD3^+^CD8^+^ T-cell lymphocyte ratio in the peripheral blood ↓TNF-*α* and IL-6 cytokine expression in serum	[183]
	Postmortem PD tissue	100 nM	**—**	↓*α*-Synuclein aggregates	[184]
*α*- and *β*-Asarone	Male C57BL/6 mice	10 mg/kg	MPTP	↑Movement behavior ↓Microglial activation	[185]
	Male C57BL/6 mice	10 mg/kg	6-OHDA	↑HVA, DOPAC, 5-HIAA levels ↑TH-positive cells in the ST region ↓JNK and p-JNK expression ↑Bcl-2 protein expression	[186]
	Sprague Dawley rats	15 mg/kg	6-OHDA	↑CAT, SOD, GPx, and GSH cerebral activity ↓TBARS activity ↑PERK/CHOP/Bcl-2/Beclin-1 pathway ↓GRP78 levels	[187]
Theaflavin	Male C57BL/6 mice	10 mg/kg	MPTP/p	↑DAT and VMAT-2 expression ↑Behavioral functions ↑CAT, SOD, GPx, and GSH cerebral activity ↓TBARS activity	[188]
	Male C57BL/6 mice	10 mg/kg	MPTP/p	↑Behavioral characterization ↑TH-positive cells in the ST region ↓Caspase-3, caspase-8, and caspase-9 activity ↓Bax expression ↑Bcl-2 protein expressions	[189]
	Male C57BL/6 mice	10 mg/kg	MPTP/p	↑Behavioral characterization ↓IL-4 and IL-10 protein expressions	[190]

## Data Availability

No data were used to support this study.

## Abbreviations

AA: Asiatic acid
AD: Alzheimer's disease
AKT: Serine/threonine protein kinase
ALP: Autophagy-lysosome pathway
ARE: Antioxidant response element
Apo: Apomorphine
BBB: Blood-brain barrier
BDNF: Brain-derived neurotrophic factor
BSA: Bovine serum albumin
CAT: Catalase
COX-2: Cyclooxygenase-2
CREB: cAMP-response element binding protein
DA: Dopamine
DAT: Dopamine transporter
DBS: Deep brain stimulation
DOPAC: 3,4-Dihydroxyphenylacetic acid
Drp1: Dynamin-related protein 1
EGCG: Epigallocatechin-3-gallate
EA: Ellagic acid
Epo: Erythropoietin
ERK: Extracellular signal-regulated kinase
ER: Endoplasmic reticulum
FA: Ferulic acid
GFAP: Glial fibrillary acidic protein
GR: Glutathione reductase
GSH: Glutathione
GSK3*β*: Glycogen synthase kinase-3 beta
GST: Glutathione S transferases
HO-1: Heme oxygenase-1
HSP-70: Heat shock protein-70
HVA: Homovanillic acid
iNOS: Inducible nitric oxide synthase
IFN*γ*: Interferon-gamma
IL-1*β*: Interleukin-1*β*
IL-4: Interleukin-4
IL-6: Interleukin-6
IL-10: Interleukin-10
Keap1: Kelch-like ECH-associated protein
LRRK2: Leucine-rich repeat kinase 2
mtDNA: Mitochondrial DNA
MAPK: Mitogen-activated protein kinase
MDA: Malondialdehyde
MPTP: 1-Methyl-4-phenyl-1,2,3,6-tetrahydropyridine
MPP^+^: 1-Methyl-4-phenylpyridinium
NADPH: Nicotinamide adenine dinucleotide phosphate
NDDs: Neurodegenerative disorders
NF-*κ*B: Nuclear factor-*κ*B
NADPH: Nicotinamide adenine dinucleotide phosphate
NO: Nitric oxide
Nrf2: Nuclear factor erythroid 2-related factor 2
PD: Parkinson's disease
PGC: Peroxisome proliferator-activated receptor gamma coactivator
PI3K: Phosphoinositide 3-kinase
PINK1: PTEN-induced kinase 1
PKC: Protein kinase C
PPAR-*γ*: Peroxisome proliferator-activated receptor gamma
PQ: Paraquat
RNS: Reactive nitrogen species
ROS: Reactive oxygen species
SN: Substantia nigra
SOD: Superoxide dismutase
SOD2: Superoxide dismutase 2
ST: Striatum
TF: Theaflavin
TH: Tyrosine hydroxylase
TNF-*α*: Tumor necrosis factor-alpha
TQ: Thymoquinone
TrkB: Tropomyosin receptor kinase B
UPS: Ubiquitin proteasome system
VMAT-2: Vesicular monoamine transporter 2
6-OHDA: 6-Hydroxydopamine.
